# Lack of phylogenetic signals within environmental niches of tropical tree species across life stages

**DOI:** 10.1038/srep42007

**Published:** 2017-02-09

**Authors:** Caicai Zhang, Jie Yang, Liqing Sha, Xiuqin Ci, Jie Li, Min Cao, Calum Brown, Nathan G. Swenson, Luxiang Lin

**Affiliations:** 1 Key Laboratory of Tropical Forest Ecology, Xishuangbanna Tropical Botanical Garden, Chinese Academy of Sciences, Menglun, China; 2 University of Chinese Academy of Sciences, Beijing, China; 3 School of Geosciences, University of Edinburgh, Edinburgh, UK; 4 Department of Biology, University of Maryland, College Park, USA

## Abstract

The lasting imprint of phylogenetic history on current day ecological patterns has long intrigued biologists. Over the past decade ecologists have increasingly sought to quantify phylogenetic signals in environmental niche preferences and, especially, traits to help uncover the mechanisms driving plant community assembly. However, relatively little is known about how phylogenetic patterns in environmental niches and traits compare, leaving significant uncertainty about the ecological implications of trait-based analyses. We examined phylogenetic signals within known environmental niches of 64 species, at seedling and adult life stages, in a Chinese tropical forest, to test whether local environmental niches had consistent relationships with phylogenies. Our analyses show that local environmental niches are highly phylogenetically labile for both seedlings and adult trees, with closely related species occupying niches that are no more similar than expected by random chance. These findings contrast with previous trait-based studies in the same forest, suggesting that phylogenetic signals in traits might not a reliable guide to niche preferences or, therefore, to community assembly processes in some ecosystems, like the tropical seasonal rainforest in this study.

Understanding the factors influencing local patterns of species co-occurrence in tropical rainforest tree communities is a crucial step towards the identification of mechanisms underlying community assembly^1^,^2^. Deterministic niche processes and stochastic neutral processes may both play substantial roles in maintaining species co-occurrence^3^,^4^, but their relative importance has proved very hard to establish^5^,^6^,^7^. In particular, while there is ample evidence that niche differentiation along environmental gradients influences species distributions and community structure at local scales^8^,^9^,^10^,^11^,^12^, the generality and ecological significance of this effect remains unclear.

Recently, phylogenetic community spatial structure has been increasingly used to illuminate such factors and therefore to make inferences about community assembly processes^1^,^13^,^14^,^15^,^16^,^17^,^18^. Phylogenetic clustering in space might indicate environmental filtering, while phylogenetic over dispersion in space might indicate inter-specific competition^14^,^18^,^19^. However, these interpretations depend on a potentially unsafe assumption that closely-related species tend to occupy more similar ecological niches than distantly-related species^2^,^16^,^20^.

In order to investigate the ecological similarity of related species, many other studies have quantified phylogenetic signals in functional traits, instead of space, to inform inferences regarding community assembly^17^,^21^,^22^. Where functional traits show a positive phylogenetic signal, meaning that closely related species display more similar traits than distantly related species, then phylogenetic relatedness has been interpreted as indicative of ecological similarity^14^,^23^. This interpretation has been further extended to cover species’ environmental niches, with phylogeny used as a guide not only to general ecological characteristics but also as a proxy for specific niche preferences^21^,^24^,^25^. The advantage of this approach is that it allows inferences to be made about complex coexistence mechanisms on the basis of relatively easily-measurable functional traits^1^,^17^,^18^,^26^. However, these inferences rely on a potentially unsafe assumption that traits are related to environmental requirements in constant and predictable ways^25^,^27^.

In order to ensure appropriate interpretation of phylogenetic information, it is therefore necessary to know more about how phylogenetic structure in space and traits relates to ecologically significant characteristics such as niche preferences. Studies that use environmental data to directly measure local-scale environmental niches and test their phylogenetic signal are comparatively rare, making it hard to draw robust, general conclusions^28^,^29^,^30^,^31^. In this study, we investigate phylogenetic signals in known environmental niches of common species of seedlings and large trees in a tropical forest dynamics plot in Xishuangbanna, southwest China. We use phylogenetic comparative analysis to quantify phylogenetic signals and determine whether local environmental niches (based on topographic and soil factors) relate to phylogenies in meaningful and consistent ways across life stages. We expect local environmental niches to have strong phylogenetic signals across life stages, with closely-related species occupying more similar local environmental niches than expected by chance. If true, this would imply that phylogenetic relatedness may be used as a proxy for ecological similarity, making phylogenetic community structure a reasonable basis for inference about community assembly processes at the local scale. Alternatively, if phylogenetic signals within environmental niches are absent or variable across life stages, then inferences regarding community assembly drawn from phylogenetic dispersion patterns may be unreliable.

## Results

### Phylogenetic signals within local-scale environmental niches

The three statistical analyses employed here gave consistent results for the extent of phylogenetic signals within environmental niches. Blomberg’s *K* statistic showed only one significant phylogenetic signal (with adult trees being phylogenetically overdispersed along the third soil PCA axis), otherwise indicating a lack of phylogenetic signal in environmental niches (Table 1). Similarly, Sankoff parsimony scores showed no significant phylogenetic signals in categorical habitat associations in either seedlings or large trees (Figs S2 and 3). Both of these findings suggest that species-habitat associations were evolutionary labile.

The Net Relatedness Index (NRI) and Nearest Taxon Index (NTI) agreed in the majority of cases (Tables 2 and 3), but did show significant phylogenetic overdispersion within the (small) group of species positively associated with the ridge habitat at seedling stage (Table 2). With this single exception, the lack of phylogenetic signals was found to hold across life stages (Tables S3).

## Discussion

Niche separation along environmental gradients is thought to play a significant role in community assembly^32^. However, this role is difficult to assess in tropical forests, where species diversity and environmental complexity often make the identification of niches extremely difficult. Consequently, a number of studies have used more easily-measured functional traits as a proxy for local-scale niche preferences^1^,^17^,^18^. Phylogenetic signals in trait occurrence are then taken as indicative of relationships between evolutionary relatedness and local-scale environmental niche preferences^1^,^18^,^21^.

However, the extent of any actual phylogenetic structure within niches has rarely been directly assessed, and inferences about community assembly processes are often, therefore, based on a largely untested assumption^33^. In the present study, we directly quantified local-scale environmental niches and measured phylogenetic relatedness within these in order to examine the reliability of phylogenies as guides to niche-based mechanisms of community assembly. Contrary to our expectations, our results demonstrated a clear lack of phylogenetic signals within environmental niches at the local scale. In other words, closely related species did not share similar environmental niches more often than expected by chance, suggesting that environmental niches are evolutionarily labile (in the Xishuangbanna 20-ha forest dynamics plot, at least). Indeed, the only statistically-significant relationships we found showed phylogenetic overdispersion, rather than conservatism, within niches. Furthermore, our results indicate lability in local-scale environmental niches both in seedlings and large trees.

It is important to view these findings in the context of previous studies that have found significant phylogenetic signals in functional traits in the same plot^1^. This earlier work was taken to support the use of phylogeny as a surrogate for niche conservatism, under the assumption that traits strongly map onto niches. Similar assumptions of phylogenetic niche conservatism have underpinned other work that has sought to improve understanding of species coexistence and community assembly processes^14^,^34^,^35^. Nevertheless, some earlier studies have suggested that niches can be evolutionarily labile rather than conserved at the local scale^36^,^37^. Our new findings add substantially to the evidence for such lability, and therefore indicate considerable scope for misinterpretation of research that assumes particular phylogeny-trait-niche relationships to make inferences about community assembly.

It is clear that phylogenetic information must be used with caution when assessing mechanisms of coexistence for which no direct data are available, especially in the case of environmental niche differentiation. While phylogenies may still provide crucial insights into the role of niche differentiation in structuring communities, more work must be done on how niche preferences vary with relatedness, whether niches may exist on previously unmeasured axes, and whether patterns of variation contribute to species coexistence^38^. The approach used here, of first identifying environmental niches and then investigating phylogenetic signals within them, is especially promising, providing a firm basis for later inferences about community assembly processes.

## Methods

### Study site

This study took place in the 20-ha Xishuangbanna Forest Dynamics Plots (FDP) located in Yunnan Province, South-Western China (21°36′N and 101°34′E). The annual mean precipitation is 1500 mm, of which 84% falls during the May-October wet season^39^ (A full description of the site’s climate and floristic composition can be found in Cao *et al*.^39^). All woody plants with ≥1 cm diameter at breast height (DBH) were measured, mapped, tagged and identified to species between 2006 and 2007^40^.

During 2010 a total of 500 seedling quadrats (2 m × 2 m) were established in the center of each 20 m by 20 m subplot in the 20-ha plot. All woody plants with height ≥20 cm and DBH < 1 cm were tagged and identified to species between January and April 2010. There were 298 woody plant species found in this first census. Seedling quadrats were censused in every subsequent year during April-May.

### The measurement of environmental niches

We used conditional probability to calculate local-scale environmental niches of each species based on four topographic factors (aspect, convexity, elevation and slope) and three soil fertility PCA axes from nine soil nutrients. Detailed information of soil data collection and measurement can be found in Hu *et al*.^41^. The three PCA axes accounted for 95% of the variation in soil nutrient availability. The conditional probability of species occurrence (E) for a given habitat variable *x*, p(E|*x*) represents the probability that a focal species exists at a selected point when the environmental variable of the point is *x*^42^. We calculated the environmental niche as the value *x* when p(E|*x*) reaches its maximum, with *x* representing the measured topographical variables and three soil fertility PCA axes.

First, the four topographic variables were used to classify the 20-ha plot into three habitats: valley (213 20 m × 20 m quadrats), slope (149 20 m × 20 m quadrats) and ridge (138 20 m × 20 m quadrats) (Fig. S1; Table S1). Torus-translation tests^8^ were then used to test for significant associations of species with each of the three habitat types, with a significance level of 0.025.

In this study, we focused on dicot tree species and excluded lianas, shrubs and palms. We separately selected seedling species (with *n* ≥ 20; 2010 census) and adult tree species (*n* ≥ 100; 2007 census) as target species, and removed two species (seedling: *Ervatamiat enuifolia* and large tree: *Walsura robusta*) not included in a previously-established molecular phylogeny for the Xishuangbanna plot^1^. This left a total of 78 species of seedlings and 127 species of adult trees used to examine phylogenetic signals, of which 64 species were common to both sets, and were therefore used to study changes in phylogenetic signals in environmental niches across life stages.

### Statistical analyses

We used three statistical approaches to look for phylogenetic signals in niche preferences. First, Blomberg’s *K* statistic was used for the continuous niche variables in our study (i.e. those calculated based on the conditional probabilities described above)^43^. This statistic compares the observed distribution of values on a given phylogenetic tree to that expected given a Brownian(random) motion model of evolution on the same phylogenetic tree. If *K* = 1, the observed niche distribution is consistent with the Brownian motion model. If *K* > 1, a positive phylogenetic signal exists in environmental niches, meaning that closely related species have more similar niches than expected under the Brownian motion model. If *K* < 1, a negative phylogenetic signal exists, and closely related species are more divergent in their niches than expected. Finally, If *K* is close to 0, there is an absence of phylogenetic signal, implying that closely related species do not have more similar niches on average than distantly related species. The *K* value is a descriptive statistic, the significance of which we determined by randomly permuting the niche values across the tips of the phylogenetic tree 999 times, to generate a null distribution of *K* values from which a *p*-value can be calculated^44^.

Second, species habitat associations were treated as categorical variables and a Sankoff parsimony score was calculated based on their distribution on the phylogeny with equal transition probabilities between habitat association types^45^. The observed parsimony score was compared to a null distribution of parsimony scores derived by permuting habitat associations types across the tips of the phylogeny 999 times. We then took a *p* value < 0.05 as indicative of closely related species having similar habitat associations.

Lastly, we calculated the Net Relatedness Index (NRI) and the Nearest Taxon Index (NTI) to measure the degree of phylogenetic relatedness within each of the four species groups (consisting of species with the same habitat preferences; valley, slope, ridge or neutral). The NRI measures the phylogenetic dispersion of an assemblage by comparing the observed mean pairwise phylogenetic distance between species in an assemblage to the null model. The NTI measures the phylogenetic dispersion of an assemblage by comparing the observed mean nearest phylogenetic neighbor distance between species in an assemblage to the null model. Assemblages here refer to groups of species associated to the same habitat type. Positive values of NRI and NTI therefore indicate that species with the same habitat preferences are phylogenetically clustered (more closely related than expected), while negative values for NRI and NTI indicate that species with the same habitat preferences are phylogenetically overdispersed (more distantly related than expected). We removed species that had negative habitat associations or positive association with more than one habitat, leaving a total of 75 seedling species and 104 adult tree species in this stage of the analysis.

## Additional Information

**How to cite this article:** Zhang, C. *et al*. Lack of phylogenetic signals within environmental niches of tropical tree species across life stages. *Sci. Rep.* **7**, 42007; doi: 10.1038/srep42007 (2017).

**Publisher's note:** Springer Nature remains neutral with regard to jurisdictional claims in published maps and institutional affiliations.

## Supplementary Material

Supplementary Information

## Figures and Tables

**Table 1:** Blomberg’s *K* statistic for environmental niches of 78 seedlings species having equal to or greater than 20 individuals and 127 species of adult trees having equal to or greater than 100 individuals.

Environmental niches	Seedlings		Large trees	
	K value	*P*	K value	*P*
Aspect niches	0.53	0.204	0.50	0.549
Convex niches	0.52	0.284	0.51	0.486
Elevation niches	0.45	0.814	0.48	0.720
Slope niches	0.48	0.565	0.51	0.472
PCA axis 1 niches	0.45	0.815	0.42	0.974
PCA axis 2 niches	0.50	0.463	0.52	0.396
PCA axis 3 niches	0.56	0.149	0.72	0.011*

*P < 0.05.

**Table 2:** The phylogenetic dispersion within each of the four species groups with the same habitat preferences at seedling stage. The analysis includes only those species with 20 or more seedlings.

Habitat preference of species group	Number of species in group	NRI	*P*	NTI	*P*
Neutral	50	1.16	0.882	1.08	0.863
Valley	12	−0.14	0.382	−0.14	0.408
Slope	8	−0.26	0.333	−0.13	0.372
Ridge	5	−2.73	0.012*	−2.69	0.014*

NRI: The Net Relatedness Index; NTI: The Nearest Taxon Index; *P < 0.05.

**Table 3:** The phylogenetic dispersion within each of the four species groups with the same habitat preferences at adult stage. The analysis includes only those species with 100 or more adults.

Habitat preference of species group	Number of species in group	NRI	*P*	NTI	*P*
Neutral	43	−1.10	0.154	−1.17	0.130
Valley	31	0.20	0.543	0.24	0.550
Slope	10	−1.02	0.148	−1.04	0.162
Ridge	20	−0.62	0.252	−0.63	0.255

NRI: The Net Relatedness Index; NTI: The Nearest Taxon Index.
