# Supplementary material for: Lack of phylogenetic signals within environmental niches of tropical tree species across life stages
Source: Sci Rep. 2017 Feb 9;7:42007. doi: 10.1038/srep42007 (PMC5299423; doi:10.1038/srep42007)
Supplement: Supplementary Information [file srep42007-s1.pdf]

Lack of phylogenetic signals within environmental niches of tropical tree species across life stages

Caicai Zhang[1,4], Jie Yang[1], Liqing Sha[1], Xiuqin Ci[1,4], Jie Li[1], Min Cao[1], Calum Brown[2], Nathan G. Swenson[1,3], Luxiang Lin[1,*]

[1]Key Laboratory of Tropical Forest Ecology, Xishuangbanna Tropical Botanical Garden, Chinese Academy of Science, Menglun, China.

[2]School of Geosciences, University of Edinburgh, Edinburgh, UK.

[3]Department of Biology, University of Maryland, College Park, USA.

[4]University of Chinese Academy of Sciences, Beijing, China.

*Correspondence and requests for materials should to be addressed to L.L. (email: linluxa@xtbg.ac.cn)

Table S1 Criterions of habitat classification for all 20 m × 20 m quadrats in the 20-ha Xishuangbanna forest dynamics plot.

Habitats	Valley	Slope	Ridge
Elevation (m)	< 779.8	all	all
Slope (degree)	all	≥ 27.14	< 27.14
Convexity (degree)	< 0	≥ 0	all

27.14 and 779.8 are median elevation and median slope respectively for all 20 m × 20 m quadrats within the plot.

Table S2 Blomberg's *K* statistic for environmental niches of 64 common species with both seedlings ≥ 20 individuals and adult trees ≥ 100 individuals.

	Seedlings		Large trees	
Environmental niches	*K* value	*P*	*K* value	*P*
Aspect niches	0.50	0.548	0.54	0.328
Convex niches	0.51	0.497	0.55	0.258
Elevation niches	0.48	0.694	0.49	0.624
Slope niches	0.51	0.448	0.57	0.201
PCA axis 1 niches	0.42	0.973	0.51	0.462
PCA axis 2 niches	0.52	0.419	0.50	0.527
PCA axis 3 niches	0.72	**0.009****	0.81	**0.002****

** $P < 0.01$

Table S3 The phylogenetic dispersion for each of the four species groups with the same habitat preferences. These analyses include only the species with 20 or more seedlings and 100 or more adult trees.

Habitat preference of species group	Seedlings			Adult trees		
	Number of species in group	NRI	*P*	Number of species in group	NRI	*P*
Neutral	39	0.74	0.773	17	-0.31	0.350
Valley	11	-0.29	0.347	19	-0.18	0.385
Slope	7	0.51	0.673	7	-1.49	0.087
Ridge	4	-1.12	0.120	9	0.10	0.527

NRI: The Net Relatedness Index; NTI: The Nearest Taxon Index

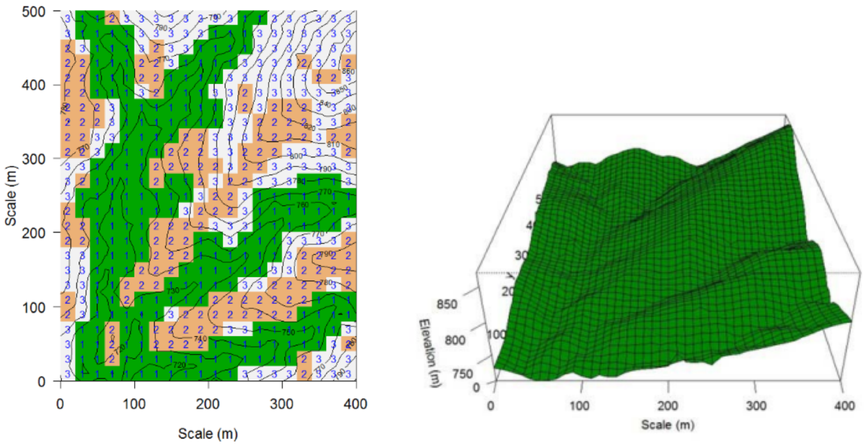

Fig S1 The spatial distribution of the three habitats types in the 20-ha

Xishuangbanna forest dynamics plot. “1” represent valley habitat; “2” represent ridge habitat; “3” represent slope habitat.

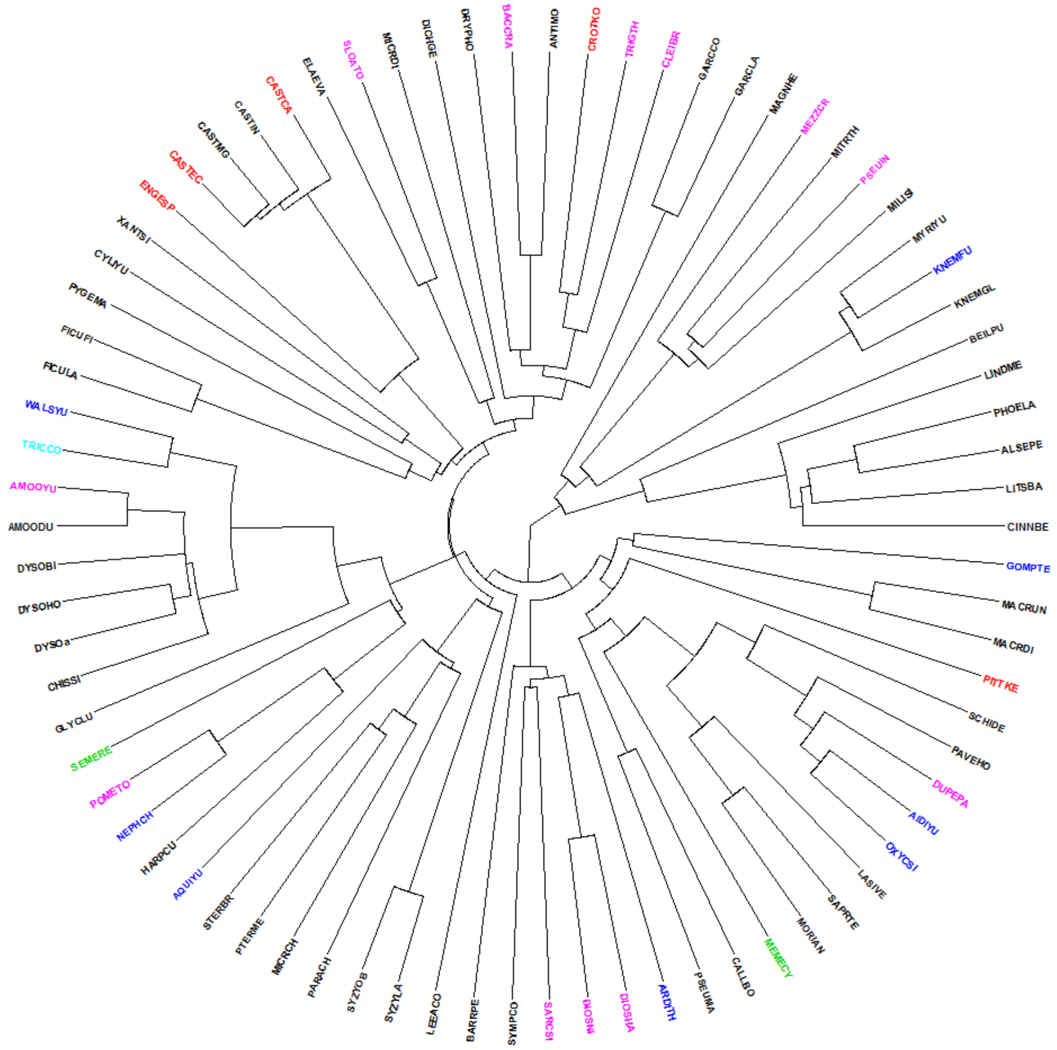

Fig S2 The phylogenetic tree constructed from DNA barcodes of the 78 species of seedlings that have different associations with three habitats in the 20-ha Xishuangbanna forest dynamics plot. “black” represent neutral species, “red” represent positive association with ridge, “blue” represent positive association with slope, “pink” represent positive association with valley, “green” represent negative with slope and “light blue” represent negative with valley.

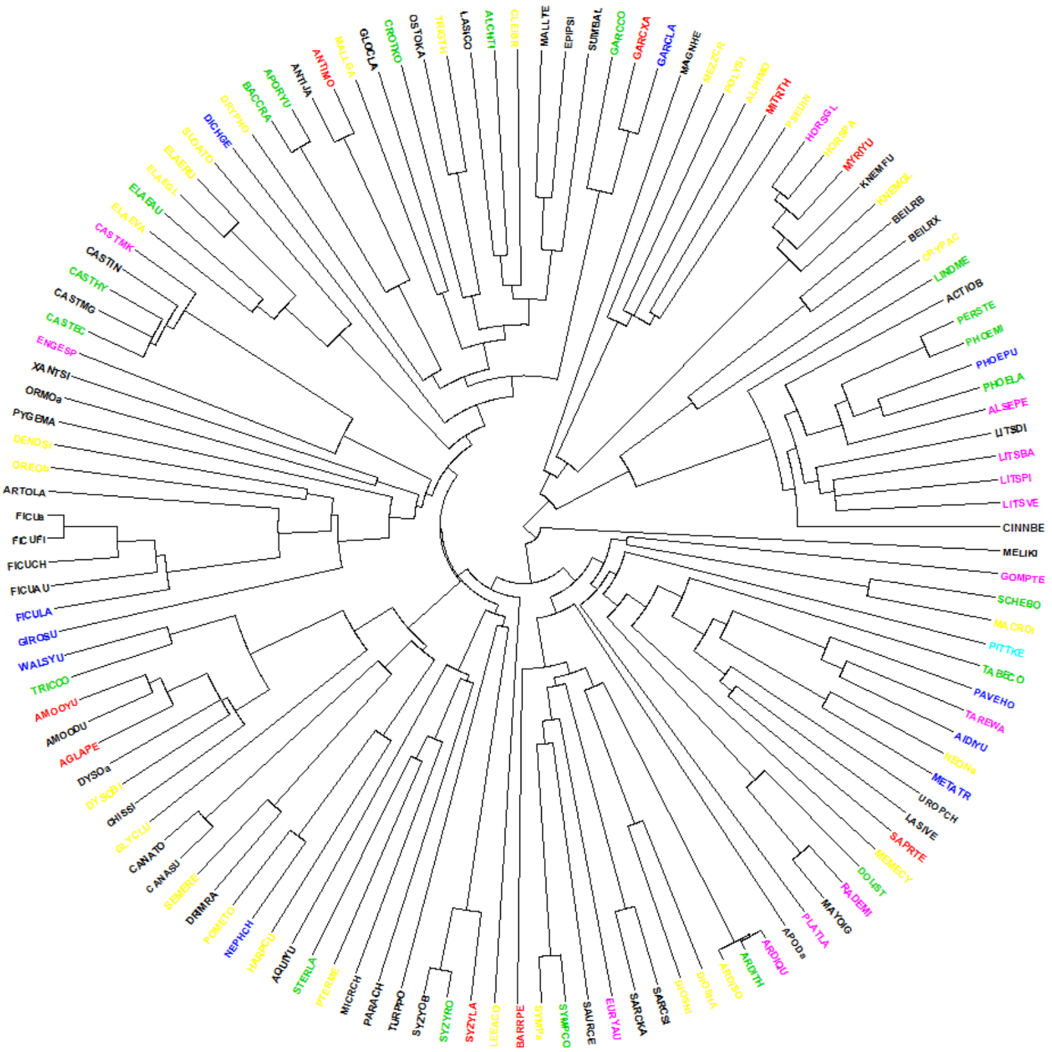

Fig S3 The phylogenetic tree constructed from DNA barcodes of the 127 species of large trees that have different associations with three habitats in the 20-ha Xishuangbanna forest dynamics plot. “black” represent neutral species, “green” represent positive association with ridge, “blue” represent positive association with slope, “yellow” represent positive association with valley, “light blue” represent positive association with slope and ridge, “red” represent negative with ridge and “pink” represent negative with valley.
